# Ultrastructural analysis of salivary glands in a phytophagous stink bug revealed the presence of unexpected muscles

**DOI:** 10.1371/journal.pone.0179478

**Published:** 2017-06-28

**Authors:** Nathaly Castellanos, Luis C. Martínez, Eder H. Silva, Adenir V. Teodoro, José Eduardo Serrão, Eugênio E. Oliveira

**Affiliations:** 1 Departamento de Entomologia, Universidade Federal de Viçosa, Viçosa-MG, Brasil; 2 Departamento de Biologia Geral, Universidade Federal de Viçosa, Viçosa-MG, Brasil; 3 Embrapa Tabuleiros Costeiros, Av. Beira Mar 3250, Aracaju–SE, Brasil; Pusan National University, REPUBLIC OF KOREA

## Abstract

The exceptional abilities of stink bugs (Hemiptera: Pentatomidae) to colonize a diverse group of plants have been attributed to the feeding behaviors and the functions of the salivary complex of these insects. Here, we describe the ultrastructure of the salivary glands of the Neotropical brown stink bug, *Euschistus heros*, which is a major component of the pentatomid pest complex on soybeans, *Glycine max*, in the neotropics. Our results revealed a salivary gland complex consisting of two lobes (i.e., anterior and posterior), with a constriction between them (i.e., the hilum), in which the salivary and accessory gland ducts are inserted. The principal gland epithelium has a single layer of cells lining an enlarged lumen filled with saliva, and these cells are cuboidal, rich in rough endoplasmic reticulum and secretory vesicles, with well-developed nuclei, all of which are typical features of protein-secreting cells. We report, for the first time in insects, the presence of a layer of muscle cells surrounding the columnar hilum epithelium. The accessory salivary gland cells are cuboidal with nuclei containing condensed chromatin and cytoplasm rich in vacuoles and rough endoplasmic reticulum, indicating the potential involvement of these glands in water transport/secretion. The lumen content of each lobe of the principal gland suggests that the lobes produce different compounds. Thus, our results suggest that the *E*. *heros* salivary complex might have unconventional mechanisms to mix/release saliva, which might help explain the polyphagous abilities of these insects.

## Introduction

In arthropods, the salivary complex plays a pivotal role in physiological processes such as extra-oral digestion, food lubrication, osmotic regulation, defense against toxic substances, pathogen elimination, and anticoagulant secretion [1–5]. In hemipteran insects, the salivary gland complex comprises a pair of principal glands and a pair of accessory glands that produce saliva [2,6–9], which is composed of a variety of substances, including water, lipids, carbohydrates and enzymes, that promote extra-oral digestion, and maintain food consumption efficiency [2,10–12].

Although morpho- and histological descriptions of salivary glands have already been conducted for some stinkbug (Hemiptera: Pentatomidae) species [2,4,9,13], these studies have mainly focused on predators, or at least zoophytophagous species, completely neglecting the stink bugs that exclusively feed on plant tissues (hereafter phytosuccivorous). This lack of knowledge is intriguing as phytosuccivorous stink bugs are key crop pests, damaging seeds and developing fruiting structures [4,14,15]. In particular, the production of soybeans, *Glycine max*, in the neotropics is threatened by a complex of stink bugs that occur throughout most of the cultivation area, with the Neotropical brown stink bug, *Euschistus heros* (F.), being the most abundant pest [4,14,16,17].

In general, stink bugs such as *E*. *heros* directly damage seed tissues, making them pinched and wrinkled, and reducing the seed oil content and the germination rate, which consequently affects grain yield and quality [14,15,18]. Furthermore, the feeding punctures caused by stinkbugs facilitate contamination by plant pathogens and may even cause physiological disturbances, such as foliar retention and delayed seed maturation [4,19,20]. Although it has been established that *E*. *heros* inserts its mouthparts into the soybean pods, lacerating the tissue while injecting saliva containing digestive enzymes to liquefy the vegetal tissue before ingestion [4,15,21], the morphophysiology of the salivary glands of *E*. *heros* have not yet been described. Thus, to enhance our understanding of the salivary gland functions in stinkbugs and to provide additional information about the bioecological interactions between *E*. *heros* and its vast group of host plants, we describe the morphology and ultrastructure of the salivary glands in these phytosuccivorous stink bugs.

## Materials and methods

All applicable international, national, and institutional guidelines for the care and use of animals were considered in the present investigation.

### Insects

Adults of *E*. *heros* were obtained from a mass-rearing colony kept under controlled laboratory conditions (27 ± 2°C, 60 ± 20% relative humidity, and an L:D photoperiod of 14:10 h) at the Laboratório de Fisiologia e Neurobiologia de Invertebrados (UFV, Viçosa-MG, Brazil). To prevent diapause, artificial lighting was maintained between 08:00 and 22:00 h, and all developmental stages of *E*. *heros* were mass-reared in plastic boxes following previously described methods [13,22–24]. Briefly, the insects were fed *ad libitum* a mixture of fresh green bean pods, *Phaseolus vulgaris* (L.); dry soybean seeds, *G*. *max* (L.); raw shelled peanuts, *Arachis hypogaea* (L.); and sunflower seeds, *Helianthus annuus* (L.), in addition to water. The plastic boxes were cleaned, and supplies were replenished at 2-day intervals. Eggs were removed from pieces of cheesecloth placed inside the mass rearing boxes and transferred to plastic Petri dishes containing a piece (ca. 3 cm) of green bean pod, and second-instar nymphs were transferred to plastic boxes and reared until they reached adulthood.

### Light and scanning electron microscopy

For the light microscopy analysis, 10 adult *E*. *heros* (i.e., five males and five females) were anesthetized at 0°C, and the salivary glands were dissected in a saline solution for insects and transferred to Zamboni's fixative solution for 24 h at 5°C. Then, the samples were dehydrated in a graded ethanol series (70°, 80°, 90°, and 95°), embedded in historesin JB4 (Electron Microscopy Sciences, Fort Washington, PA, USA), sectioned at a thickness of 3 μm in a Leica RM2255 microtome, stained with hematoxylin and eosin, and analyzed under a light microscope (Olympus BX53, Olympus Deutschland, Hamburg, Germany). For the scanning electron microscopy, the salivary glands of other group of 10 *E*. *heros* adults were dissected in a saline solution for insects (0.1 M NaCl, 0.1 M KH_2_PO_4_, 0.1 M Na_2_HPO_4_) and transferred to Zamboni's fixative solution [25] for 12 h at 5°C. The samples were subsequently dehydrated in a graded ethanol series (70°, 80°, 90°, and 99°), transferred to hexamethyldisilazane for 5 min, air dried, covered in gold (20 nm thick) and analyzed with a LEO VP1430 scanning electron microscope (Carl Zeiss, Jena, Germany).

### Detection of actin

After dissection, the salivary glands of five adult males and five adult females were transferred to Zamboni’s fixative solution for 30 min. The samples were washed three times with 0.1 M sodium phosphate buffer with 1% Triton X-100 (PBST). Then the salivary glands were incubated in dark with phalloidin-FITC conjugated 1:100 in PBST for 16 h in dark and mounted with 50% sucrose. Samples were optically sectioned at 1.5 μm until muscle was imagined in series in a confocal laser fluorescence microscope Zeiss 510 Meta (Carl Zeiss, Jena, Germany).

### Transmission electron microscopy

Salivary glands of groups of 10 *E*. *heros* adults (i.e., six males and six females) were dissected and transferred to 2.5% glutaraldehyde in a sodium cacodylate buffer (0.2 M, pH 7.2) containing 0.2 M sucrose for 4 h at room temperature. Then, the principal salivary gland was divided into its anterior lobe and posterior lobe, and the accessory gland was isolated. The samples were post-fixed in 1% osmium tetroxide for 2 h in the same buffer at room temperature followed by washing in buffer and dehydration in a graded ethanol series (70°, 80°, 90°, and 99°). The samples were embedded in LR White resin (London Resin Company Ltd.), and ultrathin sections (80–90 nm) were obtained with a glass knife in a Sorvall MT2-BMT2-B ultra-microtome (Sorvall Instruments, Wilmington, DE, USA). The sections were then stained with 1% aqueous uranyl acetate and lead citrate [26] followed by analysis with a Zeiss EM 109 transmission electron microscope (Carl Zeiss, Jena, Germany).

## Results

### Anatomy and histology

**Fig 1:**
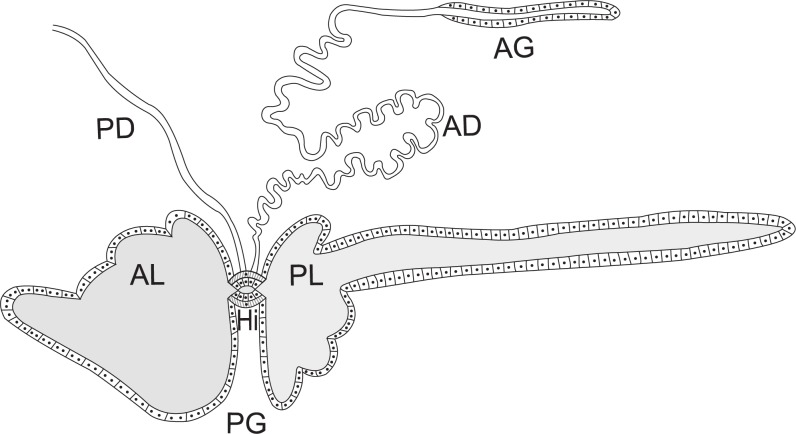
Schematic diagram of the salivary system of *E*. *heros* showing the epithelium of the principal gland (PG) with the anterior lobe (AL) and posterior lobe (PL), the hilum (Hi) and associated muscle fibers, the principal salivary duct (PD) and the duct of the accessory gland (AD) connected to the accessory gland (AG). Not drawn to scale.

**Fig 2:**
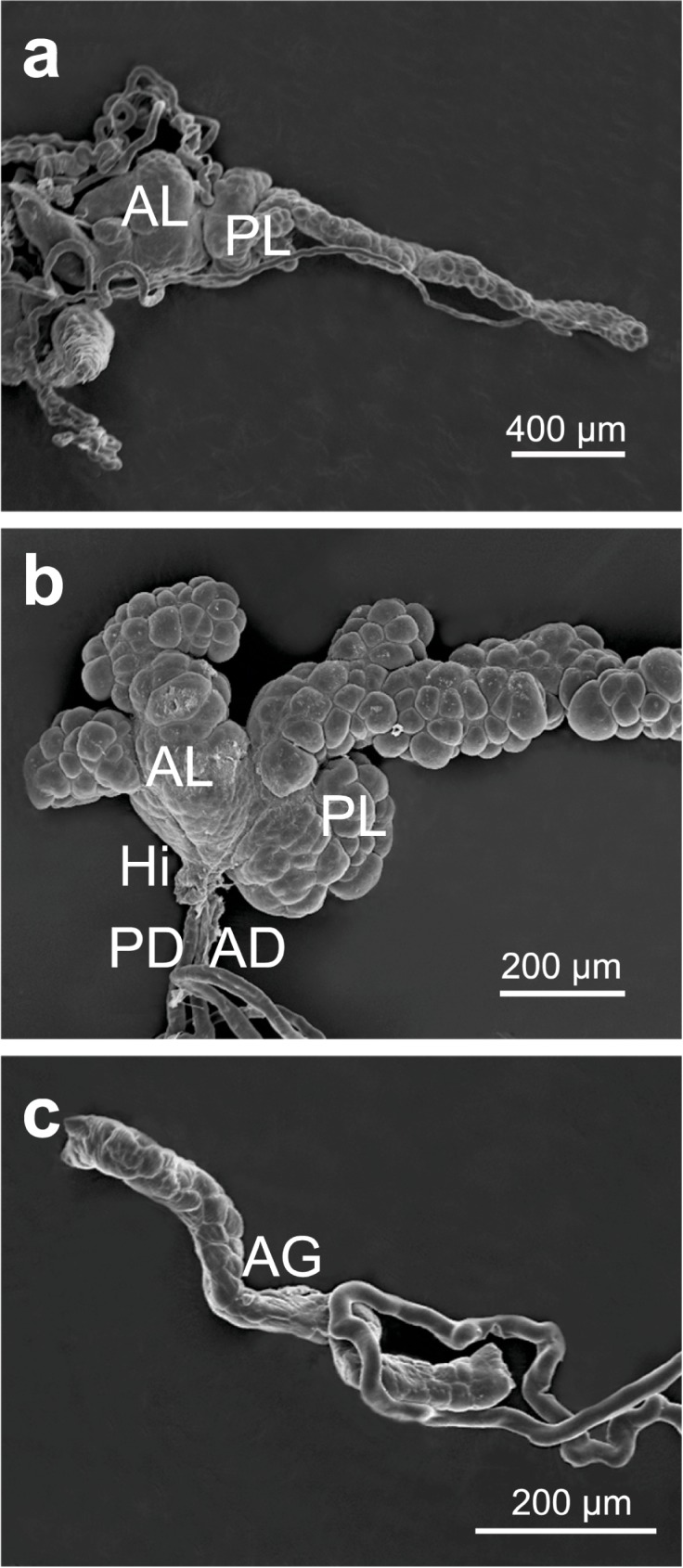
Scanning electron micrographs of the salivary glands of *E*. *heros*. (**a**): General view showing the principal salivary gland with the anterior lobe (**AL**) and the posterior lobe (**PL**) and the hilum (**Hi**) between the lobes. (**b**): Detail of the hilum (**Hi**) between the **AL** and **PL** with the ducts of principal gland (**DP**) and the accessory gland (**DA**). (**c**): Detail of the accessory salivary gland (**AG**).

The salivary gland complex of *E*. *heros* consisted of a pair of principal salivary glands and a pair of tubular accessory salivary glands (Figs 1 and 2). Both gland types were translucent when subjected to interact with physiological solution for insects. The salivary glands were located in the thoracic region, extending from the prothorax to the metathorax. The principal salivary glands were bilobed and with striking differences in size and form (Fig 2A). While the anterior lobe was smaller and having a semi-oval with four irregular projections of different sizes, the posterior lobe was elongated, presented oval shape with small projections in the proximal region, exhibiting the terminal process at the posterior end (Fig 2A). Between the anterior and posterior lobe there was a narrow region known as the hilum, in which the ducts of the principal and accessory salivary glands were inserted (Fig 2B). The principal salivary duct was connected with the salivary duct of the other principal salivary gland to form a single salivary duct that opened in the mouthpart stylet inside the head. The accessory salivary duct was long and exhibited regular U-shaped folds (Fig 2C). The final portion of the accessory salivary duct was the accessory gland. The accessory salivary glands exhibited tubular and narrower shapes than the principal salivary glands (Fig 2C).

**Fig 3:**
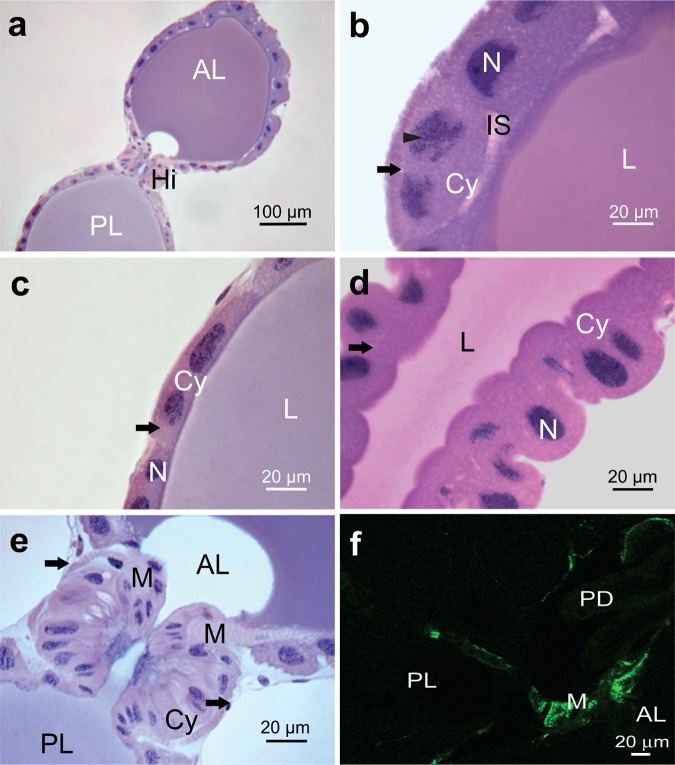
Principal salivary gland of *E*. *heros*. (**a**): General view showing the main salivary gland with the anterior lobe (**AL**), posterior lobe (**PL**), and the hilum (**Hi**) between the lobes. (**b**): Anterior lobe showing cuboidal epithelium with two well-developed nuclei (**N**) with nucleolus (arrowhead) and cytoplasm with several vesicles (**arrow**). Note the basophilic gland content in the lumen (**L**) and the enlarged intercellular space (**IS**). (**c**): Anterior portion of the posterior lobe showing squamous epithelium with two elongated nuclei (**N**) and cytoplasm (**Cy**) with multiple vesicles (**arrow**). The epithelium lines the slightly basophilic lumen (**L**) and is surrounded by a peritoneal sheath (**PS**) formed by squamous cells. (**d**): Terminal process of the posterior lobe showing cuboidal epithelium with some cells containing two nuclei (**N**) and basophilic cytoplasm (**Cy**) with several vesicles (**arrow**). The epithelium lines the slightly basophilic lumen (**L**). (**e**): Detail of the hilum between the anterior lobe (**AL**) and the posterior lobe (**PL**) showing cuboidal cells with elongated nuclei (**N**) and acidophilic cytoplasm (**Cy**). The epithelium of the hilum is surrounded by striated muscle fibers (**M**) and a peritoneal sheath (**arrow**). (**f**): Confocal micrograph of an optical slice of the region of the hilum showing actin filaments (green) in the associated muscle (M).

The epithelium of the anterior lobe of the principal salivary gland had a single layer of cuboidal cells with one or two well-developed irregular (amoeba-shape) nuclei with a predominance of decondensed chromatin (Fig 3A and 3B) and an evident nucleolus (Fig 3B). The cytoplasm was basophilic and showed a homogenous aspect with some of the vesicles, and the lumen content was homogenous and basophilic (Fig 3B). The epithelium of the initial portion of the posterior lobe of the principal salivary gland had a single layer of squamous cells with two elongated nuclei with decondensed chromatin and one or two nucleoli (Fig 3A and 3C). The cytoplasm was acidophilic with few vesicles, and the epithelial cells of the final portion were cuboidal with one or two spherical nuclei with granulated chromatin and one or two nucleoli (Fig 3D). The cytoplasm was basophilic with small vesicles, and the luminal content was slightly basophilic (Fig 3D).

**Fig 4:**
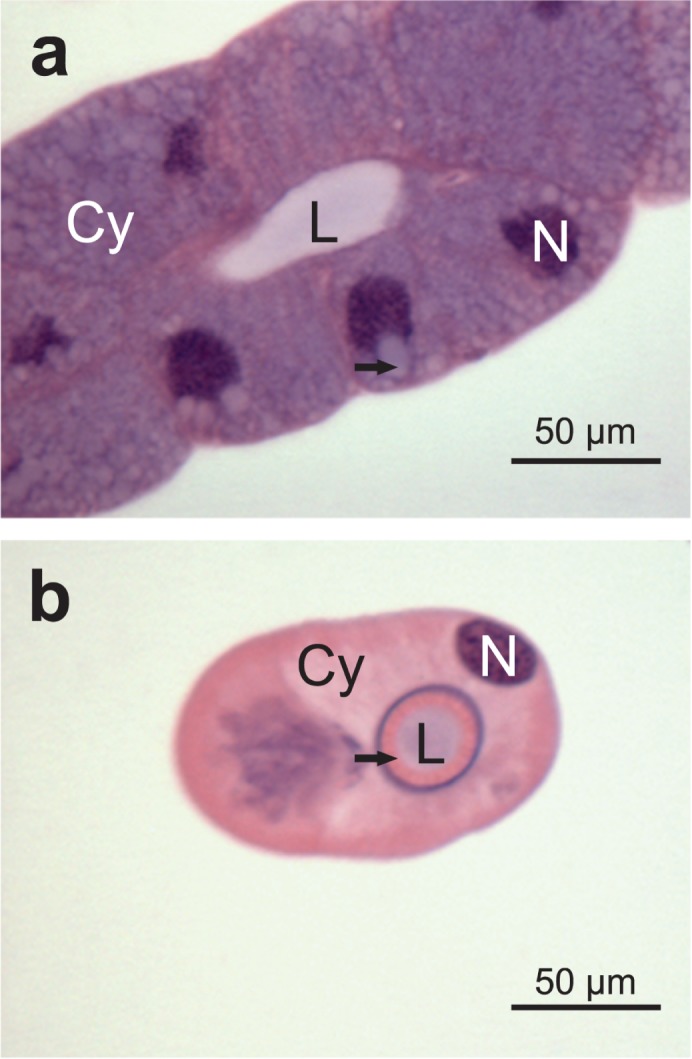
Histological sections of the accessory salivary gland of *E*. *heros*. (**a**): Secretory epithelium showing cuboidal cells with basal nuclei (**N**) and cytoplasm with several vacuoles (**arrow**). Note the narrow lumen (**L**). (**b**): Duct of the accessory salivary gland showing squamous cells with acidophilic cytoplasm (**Cy**), spherical nuclei (**N**), and a thin layer of cuticle (**arrow**) surrounding the lumen (**Lu**).

Externally, the principal salivary glands were coated with a peritoneal sheath formed by flattened cells with elongated nuclei (Fig 3A). The hilum region was between the two lobes of the principal glands (Fig 3A) with a single layer of columnar cells containing elongated nuclei with condensed chromatin (Fig 3E). The cytoplasm was acidophilic in the apical portion of the cell and slightly basophilic in the basal portion (Fig 3E), and the hilum epithelium was associated with a layer of muscle cells (Fig 3E) which was phalloidin positive (Fig 3F). The salivary duct was formed by a single layer of flattened cells with the apical portion lined with a thin cuticle. In these cells, the cytoplasm was acidophilic, and the nucleus was spherical or pleomorphic with condensed chromatin. The epithelium of the accessory salivary glands was formed by a single-layer of cuboidal cells lining a narrow lumen; the content of the lumen was stained with hematoxylin and eosin (Fig 4A). The duct of the accessory salivary glands was composed by flattened cells lined with a thin cuticle (Fig 4B).

### Ultrastructure

**Fig 5:**
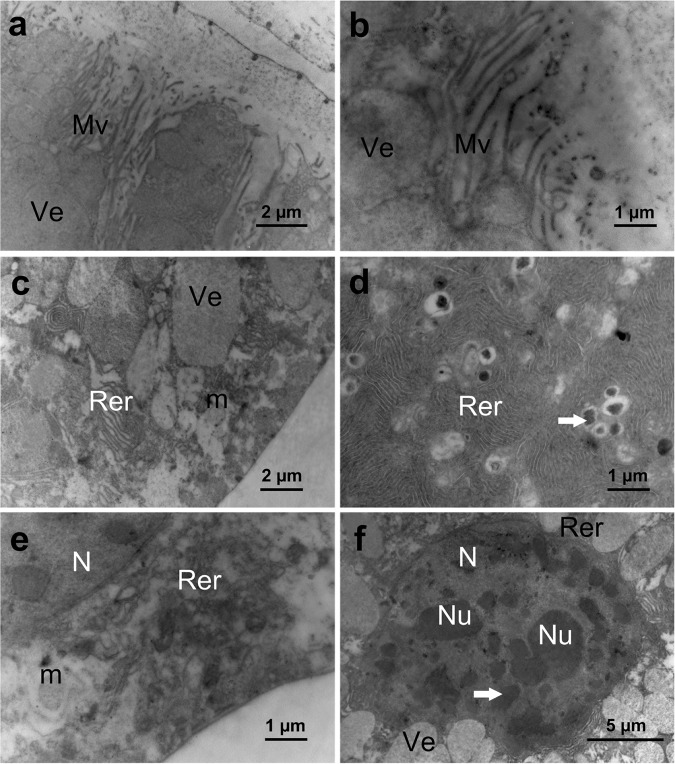
Transmission electronic micrographs of secretory cells of the anterior lobe of the principal salivary gland of *E*. *heros*. (**a**): Apical cell region showing contact between the electron-dense gland content and the cell microvilli (**Mv**). Note the gland lumen (L) with secretion (Se). (**b**): Apical cytoplasm rich in electron-lucent vesicles (**Ve**) and short microvilli (**Mv**). (**c**): Basal cell region with well-developed mitochondria (**m**), vesicles (**Ve**) and rough endoplasmic reticulum (**Rer**). (**d**): Median cell region with numerous secretion granules (**arrow**) and rough endoplasmic reticulum (**Rer**) cisterns in a concentric array. (**e**): Details of the basal cell region showing several mitochondria (**m**), rough endoplasmic reticulum (**Rer**) and the nucleus (**N**). (**f**): Nucleus (**N**) with two well-developed nucleoli (**Nu**) and condensed chromatin (**arrow**).

In the anterior lobe of the principal salivary glands, the apical surface of the secretory cells exhibited a few short microvilli (~2.0 μm long) (Fig 5A), and the lumen secretion exhibited a heterogeneous composition with an electron-lucent area in the center and becoming granular and electron-dense near microvilli (Fig 5B). The cytoplasm was rich in rough endoplasmic reticulum and vesicles with electron-lucent content (Fig 5A and 5C). The basal portion of the secretory cells had short plasma membrane infoldings forming extracellular canals associated with mitochondria and rough endoplasmic reticulum (Fig 5C), of which the cisterns formed stacks and concentric arrays (Fig 5C). In the perinuclear portion, the rough endoplasmic reticulum cisterns surrounded vesicles with electron-lucent content or small electron-dense granules characteristic of glycogen deposits and myelin-like structures (Fig 5D and 5F). Mitochondria were numerous and well developed with parallel tubular cristae (Fig 5D and 5F), and the nucleus was ovoid with an irregular surface (Fig 5F). The nucleus showed two large nucleoli, around which the chromatin was predominantly decondensed with clumps of condensed chromatin (Fig 5F).

**Fig 6:**
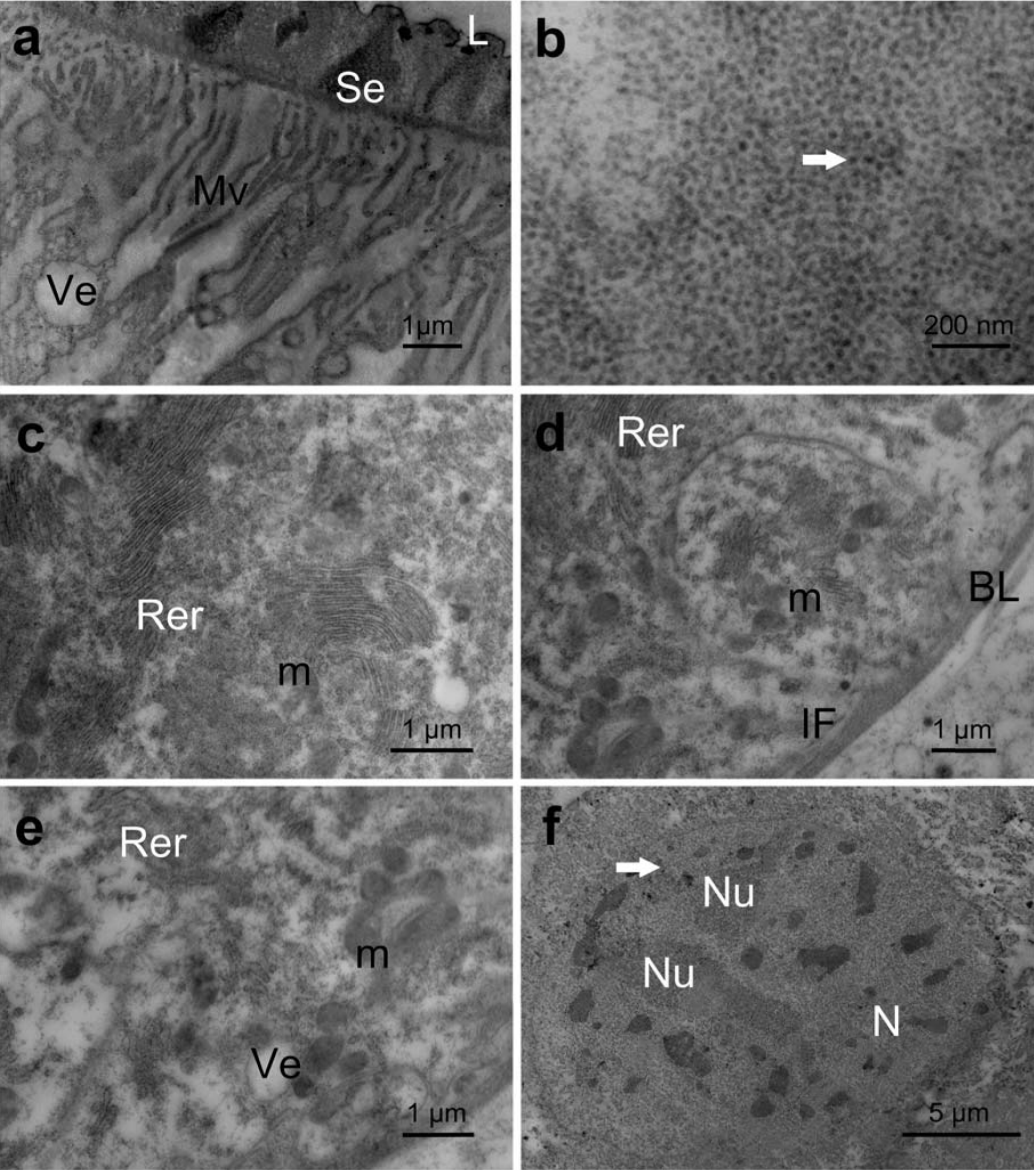
Transmission electronic micrographs of the secretory cells of the posterior lobe of the principal salivary gland of *E*. *heros*. (**a**): Apical cell region showing short microvilli (**Mv**), vesicles (**Ve**) and electron-dense gland content similar to secretion (Se) into the gland lumen (L). (**b**): Median cell region with numerous secretion granules (**arrow**). (**c**): Median cell region with numerous mitochondria (**m**) and rough endoplasmic reticulum (**Rer**). (**d**): Basal cell region showing the basal membrane infoldings (**IF**) associated with electron-dense basal lamina (BL). (**e**): Details of the basal cell region showing several mitochondria (**m**), rough endoplasmic reticulum (**Rer**) and vesicles (**Ve**). (**f**): Nucleus (**N**) with two well-developed nucleoli (**Nu**) and condensed chromatin (**arrow**).

In the posterior lobe of the main salivary gland, the apical region of the secretory cells was similar to that found in the anterior lobe, with short and narrow microvilli (~4 μm long) (Fig 6A). The secretion stored in the lumen of the gland had a heterogeneous appearance with an electron-dense granular content in the area near the microvilli and electron-lucent content in the center (Fig 6A). The apical and perinuclear cytoplasm regions were electron dense due to the presence of a large amount of rough endoplasmic reticulum, secretory vesicles with homogeneous electron-lucent content and small electron-dense granules similar to glycogen (Fig 6A–6C). The basal portion of the secretory cells of the posterior lobe had some cisterns of rough endoplasmic reticulum and mitochondria, which were associated with short basal membrane infoldings (Fig 6D). The cistern of the rough endoplasmic reticulum of both the basal and perinuclear regions arrayed in stalks (Fig 6C and 6D). The mitochondria were numerous and well developed with parallel tubular cristae (Fig 6E). The nuclei of these cells were well developed with a predominance of decondensed chromatin and well-developed nucleoli (Fig 6F).

**Fig 7:**
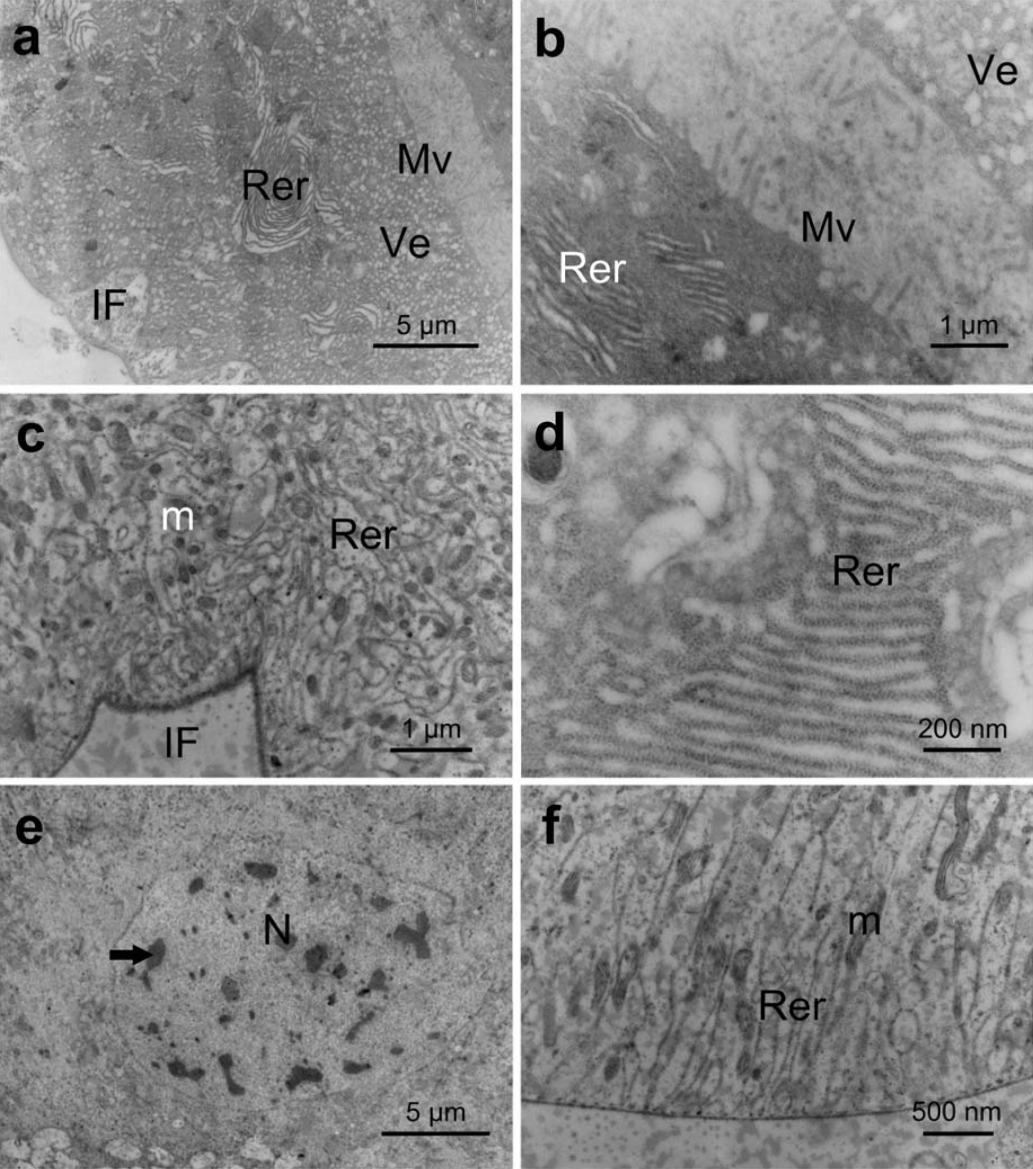
Transmission electronic micrographs of the secretory cells of the accessory salivary gland of *E*. *heros*. (**a**): General view of a secretory cell showing the apical region with short microvilli (**Mv**) and vesicles (**Ve**), the median cell region rich in rough endoplasmic reticulum (**Rer**) and the basal region with basal membrane infoldings (**IF**) (**b**): Apical cell region showing vesicles (**Ve**) and rough endoplasmic reticulum (**Rer**). (**c**): Basal cell region showing the basal membrane infoldings (**IF**) associated with several mitochondria (**m**) and rough endoplasmic reticulum (**Rer**). (**d**): Median cell region with rough endoplasmic reticulum (**Rer**) cisterns in a stalked array. (**e**): Well-developed nucleus (**N**) with condensed chromatin (**arrow**). (**f**): Details of the basal cell region showing several mitochondria (**m**) with parallel tubular cristae.

The cytoplasm of the secretory cells of the accessory salivary glands had small vacuoles with electron-lucent content, rough endoplasmic reticulum and mitochondria (Fig 7A–7C). The apical surface had short and narrow microvilli (~0.5 μm) (Fig 7B), and the apical cytoplasm showed few pleomorphic mitochondria. The basal region of the cell was characterized by plasma membrane infoldings, which were associated with mitochondria (Fig 7C) and extended throughout 1/4 of the cell (Fig 7A). The rough endoplasmic reticulum was abundant in the perinuclear region in stacked and concentric arrays (Fig 7A and 7D). The nucleus was ovoid and large with irregular surface (Fig 7E), and its nucleolus was predominantly decondensed with clumps of condensed chromatin (Fig 7E). The mitochondria were numerous and well developed with parallel tubular cristae (Fig 7F).

## Discussion

The morphological diversity of the salivary glands of hemipteran species may reflect their different feeding habits (i.e., zoophagous, phytophagous, zoophytophagous and hematophagous) [11,12,27,28]. Our results revealed that *E*. *heros* has a salivary system consisting of a pair of bilobed principal salivary glands and tubular accessory salivary glands, which are anatomically similar to those described for other stink bugs [7,29,30]. Furthermore, we report, for the first time, the presence of muscles in the hilum of insect salivary glands.

The presence of muscles in the salivary glands may be associated with the mechanisms underlying saliva transport control, and the muscles probably contribute to the regulation of the secretions released from the different compartments of the salivary glands [1]. In the salivary glands of other species of Hemiptera, such as *Peregrinus maidis* Ashmead (Delphacidae) [6] and *Mahanarva fimbriolata* Stål (Cercopidae) [1], muscles have been reported to externally line the epithelium and salivary ducts. In cockroaches [31–33] and termites [34], the saliva-releasing mechanism functions with the participation of some thorax muscles. Although not addressed in details here, it has been shown in other insects that the actions of the muscles reported in insect salivary glands have been described to be neurally controlled via direct innervation from the subesophageal ganglion and/or the stomatogastric nervous system [5,35,36].

Although further experiments are needed before firm conclusions can be drawn, it is reasonable to think that the muscles reported at the hilum of the *E*. *heros* salivary glands might functionally contribute to control the mixture of compounds produced in the anterior and posterior glandular lobes, and to the quantity of saliva released from the principal and accessory glands. Such controlling mechanisms are needed as these insects must adjust their saliva composition according to their age as well as the physical-chemical characteristics of their food [35,37,38].

As previously demonstrated in other insects [2,9,13,39], the occurrence of glandular epithelium with columnar or cuboidal cells in the three regions (i.e., anterior and posterior lobes of the principal salivary gland and the accessory salivary gland) of the *E*. *heros* salivary system suggest that all of these regions produce saliva. Furthermore, our results show that the secretory cells of the anterior and posterior lobes of the principal salivary glands of *E*. *heros* have a cytoplasm rich in rough endoplasmic reticulum and vesicles, which indicates protein synthesis in these regions [2,9,13,40]. Lending more evidence to this hypothesis, the presence of large irregular nuclei with decondensed chromatin exhibit high metabolic activity [39]. The ramifications of the nuclei cause an incremental increase of the surface area and facilitate the material transport between the nucleus and cytoplasm for protein synthesis [39,40]. However, the presence of narrow and short basal plasma membrane infoldings associated with few mitochondria in the secretory cells of the anterior and posterior glandular lobes of *E*. *heros* suggest low substance transport from the hemolymph, as in *P*. *distinctus* [9]. Additionally, we demonstrated that the secretory vesicles in the anterior and posterior glandular lobes differ in shape, size and electron density, indicating that different compounds are synthesized in these regions [5,40]. This differentiation between the lobes was also observed in other hemipterans independent of the feeding habits, including generalist predators and phytophagous heteropterans [5,41].

Based on our results, it is reasonable to think that the epithelium of the accessory glands of *E*. *heros* plays an important role in the transport of substances from the hemolymph. The presence of multiple folding of the basal membrane, large numbers of electron-lucent vesicles, few electron-dense vesicles, and numerous mitochondria are usually associated with cells engaged in passive water transport [27]. Furthermore, as previously described in the salivary complex of the stink bug predators *B*. *tabidus* [13], *P*. *nigrispinus* [2] and *P*. *distinctus* [40], the accessory salivary glands of *E*. *heros* are tubular with a narrow lumen and a duct opening in the hilum between the two lobes of the principal salivary glands. These factors makes the storage in the lumen unlikely and suggests that secretions produced in accessory glands are transported to the lumen of the principal salivary gland, as it has been previously reported by[2,9].

The epithelium of the salivary and accessory ducts is lined by a cuticle, indicating the need for an efficient separation of the lumen content and the hemolymph to protect against autointoxication or even prevent the secretions being altered by chemicals from the hemolymph [1,39]. Other potential relevant roles of the accessory salivary glands refers to their contributions on the control of osmoregulatory functions and constitution of the saliva aqueous portion [1,5]. Although of these evidences, the secretion of proteins by the *E*. *heros* accessory salivary glands could not be discarded, because well-developed rough endoplasmic reticulum and protein granules have been reported in the accessory gland of other hemipteran insects (i.e., *P*. *nigrispinus*) [2].

Due to the phytophagous feeding habits of *E*. *heros*, the secretory cells of the anterior lobe of the principal salivary gland may play a role in the production of proteins that aid in extra-oral digestion before ingestion [7,10], whereas the secretory cells of the posterior lobe may produce other substances, such as carbohydrates, lipids, and proteins, as well as transport water, all of which contribute to the final composition of the saliva [2,10,42]. Thus, the present study demonstrates that the salivary complex of *E*. *heros* has a high capacity to produce saliva with varied compositions, which might explain the ability of these phytophagous insects to feed on a variety of plant species. Furthermore, the presence of unexpected muscles associated with the hilum of the salivary glands suggests that they might use unconventional strategies to control saliva production and/or release. Further experiments aimed at investigating the morphophysiology of the unexpected muscles associated with the hilum of the salivary glands will contribute to our understanding of salivary gland function in the feeding behavior of these polyphagous herbivores.
